# In this month’s *Bulletin*

**DOI:** 10.2471/BLT.15.000815

**Published:** 2015-08-01

In the editorial section, Gyambo Sithey et al. (514) discuss Bhutan’s continued use of gross national happiness as a measure of development.

In the news section, Andrei Shukshin reports on WHO’s efforts to boost health worker capacities in Ukraine (517–518). Fiona Fleck interviews Pascal Michel on the use of space technologies for public health (519–520).

## Argentina, Brazil, Chile, Haiti, Honduras, Mexico and Peru

### Cell counts and viral loads

Pablo F Belaunzarán-Zamudio et al. document infrequent monitoring of patients receiving antiretrovirals. (529–539)

## Cameroon

### From policy to reality

Jacques DA Ndawinz et al. propose measuring time-to-treatment for HIV. (521–528)

## Ghana

### Hidden obstacles to mental health

Helen Jack et al. highlight the difficulties of providing mental health care. (587–588)

## Japan

### *Campylobacter, Salmonella* and *E. coli*

Yuko Kumagi et al. adjust national estimates of major foodborne diseases.(540–549)

## South Sudan

### Safe water for refugees

Syed Imran Ali et al. study****water quality in refugee camps. (550–558)

## Global

### The true cost of health care

Anthony L Bui et al. review all available national health expenditure data. (566–576)

### Surviving accidents and emergencies

Ziad Obermeyer et al. analyse the provision of emergency care in low-resource settings.(577–586)

### Cash transfers for climate change

Frank Pega et al. describe cash transfers designed to mitigate the health effects of climate change. (559–565)

